# Epstein-Barr Virus, Human Papillomavirus and Mouse Mammary Tumour Virus as Multiple Viruses in Breast Cancer

**DOI:** 10.1371/journal.pone.0048788

**Published:** 2012-11-19

**Authors:** Wendy K. Glenn, Benjamin Heng, Warick Delprado, Barry Iacopetta, Noel J. Whitaker, James S. Lawson

**Affiliations:** 1 School of Biotechnology and Biomolecular Sciences, University of New South Wales, Sydney, Australia; 2 Douglass, Hanly, Moir – Pathology, Sydney, Australia; 3 University Department of Surgery, University of Western Australia, Perth, Australia; Ecole Normale Supérieure de Lyon, France

## Abstract

**Background:**

The purpose of this investigation is to determine if Epstein Barr virus (EBV), high risk human papillomavirus (HPV), and mouse mammary tumour viruses (MMTV) co-exist in some breast cancers.

**Materials and Methods:**

All the specimens were from women residing in Australia. For investigations based on standard PCR, we used fresh frozen DNA extracts from 50 unselected invasive breast cancers. For normal breast specimens, we used DNA extracts from epithelial cells from milk donated by 40 lactating women. For investigations based on *in situ* PCR we used 27 unselected archival formalin fixed breast cancer specimens and 18 unselected archival formalin fixed normal breast specimens from women who had breast reduction surgery. Thirteen of these fixed breast cancer specimens were ductal carcinoma *in situ* (*dcis*) and 14 were predominantly invasive ductal carcinomas (*idc*).

**Results:**

EBV sequences were identified in 68%, high risk HPV sequences in 50%, and MMTV sequences in 78% of DNA extracted from 50 invasive breast cancer specimens. These same viruses were identified in selected normal and breast cancer specimens by *in situ* PCR. Sequences from more than one viral type were identified in 72% of the same breast cancer specimens. Normal controls showed these viruses were also present in epithelial cells in human milk – EBV (35%), HPV, 20%) and MMTV (32%) of 40 milk samples from normal lactating women, with multiple viruses being identified in 13% of the same milk samples.

**Conclusions:**

We conclude that (i) EBV, HPV and MMTV gene sequences are present and co-exist in many human breast cancers, (ii) the presence of these viruses in breast cancer is associated with young age of diagnosis and possibly an increased grade of breast cancer.

## Background

The purpose of this investigation is to determine if Epstein Barr virus (EBV), high risk human papillomavirus (HPV), and mouse mammary tumour viruses (MMTV) co-exist in some breast cancers.

For many decades these and other oncogenic viruses have been hypothesised as having potential causal roles in breast cancer [1]. The main candidate viruses include EBV, high risk HPV and MMTV [1]. Bovine leukemia virus (BLV) is a less well known virus that has been shown to be associated with human breast cancer [2]. Each of these viruses has known oncogenic potential and has been identified in normal and malignant human breast tissues. However, the identification of HPV, MMTV and EBV in breast tumours has been challenged [3]–[5]. The reasons for these challenges vary and include failure of some investigators to identify viral sequences in breast tumours and breast cancer cultured cell lines, concern about contamination of analyses based on polymerase chain reaction (PCR) techniques, and concern that outcomes based on immunohistochemistry may be non-specific and due to cross reacting proteins.

### EBV and breast cancer

The most specific evidence for an association between EBV and breast cancer, is the identification of EBV gene sequences within breast tumours. We have identified 32 published studies concerning EBV in breast cancer (Table S1). Twenty five of the studies were primarily based on standard (liquid phase) polymerase chain reaction (PCR) analysis; EBV sequences were identified in 21 of these studies. Five of the studies were primarily based on immunohistochemistry (IHC) and/or *in situ* hybridization techniques (ISH); EBV was not identified in any of these studies. Only three studies used normal breast tissues from normal (non-cancer) women as controls; EBV was not identified in normal breast tissues in two of these studies, and EBV was identified in 23% of cancer and 35% of normal specimens in the third study [6]. Evaluation of EBV in breast cancer is difficult because of the extremely low EBV viral loads. Despite these difficulties, it has become increasingly accepted by workers in this field that EBV can be identified in breast cancer specimens by specific PCR techniques [7].

Many of the studies of EBVs in breast cancer have not been conclusive because whole tumour studies based on standard PCR techniques cannot distinguish between cancer cells and infiltrating lymphocytes. Studies such as those by Bonnet *et al* [8], which used immunohistochemical techniques with antibodies against the EBV EBNA1 protein to identify EBV in breast cancer cells, have been discounted because these antibodies cross react with other proteins. However, in a definitive study of EBV and breast cancer, Fina *et al* [9] used a combination of PCR, in situ hybridization (ISH) and micro-dissection techniques to demonstrate the location of EBV sequences in breast tumours.

Normal breast epithelial cells can be infected by direct contact with EBV containing lymphatic cultured cell lines [10]. In addition, EBV has been identified in human milk and transfection of EBV DNA stimulates the growth of human milk cells [11], [12]. However, EBV has not been identified in breast cancer derived cultured cell lines.

The oncogenic mechanisms for EBV in epithelial cells (leading to naso-pharyngeal and other carcinomas) appear to differ from that for lymphatic cells (leading to lymphomas) [13]. EBV is known to promote epithelial cell growth [14]. Lin *et al* [15] have demonstrated that EBV encoded BARFO (a major EBV gene product) promotes the oncogenic activity of cultured breast cancer cells through activation of HER2 and HER3 signaling cascades.

EBV is accepted as a major contributor to Hodgkin lymphoma and there are striking and highly significant correlations between the incidence of Hodgkin lymphoma and breast cancer which suggests that EBV may also contribute to some breast cancers [16].

### HPV and breast cancer

High risk HPV types 16, 18 and 33 have been identified in breast cancers from widely different populations [17]. The prevalence of HPV positive breast cancer in these studies varies widely. Women with HPV positive breast cancer are significantly younger than those with HPV negative breast cancer with the implication that young sexually active women who have high HPV cervical infection rates which may be associated with young age breast cancer [18].

### MMTV and breast cancer

MMTV-like virus has been a major suspect as a cause of some human breast cancers for over 50 years. This is because MMTV is the well established etiologic agent of mammary tumours in field and experimental mice and MMTV gene sequences have repeatedly been identified in human breast cancers [19], [20], [21]. The biology of MMTV in mice is almost exactly mirrored in humans [1]. The main difference is the endogenous transmission of MMTV in mice which does not appear to occur in humans. We have recently demonstrated that MMTV gp52 protein and *Wnt1* are highly expressed in some MMTV-like virus positive breast tumours, and that many MMTV positive invasive ductal carcinoma specimens have histological similarities to MMTV positive mouse mammary tumours [19]. In addition we have identified MMTV-*env* sequences in human milk from 5% of normal lactating mothers [22]. MMTV can infect, integrate and multiply in human breast epithelial cancer cell lines [1].These observations are compatible for a role of MMTV in some human breast cancers.

Here we report the identification of EBV sequences in 34 (68%), high risk HPV sequences in 24 (48%), and MMTV sequences in 39 (78%) of DNA extracted from 50 fresh frozen invasive breast cancer specimens. These same viruses were identified in selected archival fixed normal and breast cancer specimens by *in situ* PCR. Sequences from more than one viral type were frequently identified in the same breast cancer and normal breast specimens.

## Methods

### Ethics

This project has formal ethics approval by the University of New South Wales Human Research Ethics Committee – number HREC HC11421. De-identified archival specimens were used in this study. With respect to some specimens there has been a long period (up to 15 years) between the date of specimen collection and use of the specimens for research purposes. Because of the de-identification, the need to avoid causing unhelpful anxiety among the donors and the many years since collection of some specimens, our IRB waived the need for informed consent for use of these archival specimens. Patient approval for research on more recent tissues, collected for clinical reasons, has been obtained.

Ethics approval for human milk collection was formally approved by the University of New South Wales, Australia, Human Research Ethics Committee (HREC 05163). All donors of human milk gave informed, prior consent.

### Specimens

All the specimens were from women residing in Australia. For investigations based on standard PCR, we used fresh frozen DNA extracts from 50 unselected invasive breast cancers. Forty normal DNA controls were obtained from epithelial cells in human milk from normal lactating women who had no history of breast cancer. For investigations based on *in situ* PCR we used a different series of 27 unselected archival formalin fixed breast cancer specimens. Thirteen of these fixed breast cancer specimens were ductal carcinoma *in situ* (*dcis*) and 14 were predominantly invasive ductal carcinomas (*idc*). Eighteen unselected archival formalin fixed normal breast specimens from women who had breast reduction surgery were used as a comparative group.

#### Genomic DNA preparation

Previously described protocols were used to extract genomic DNA from the 50 invasive breast cancer specimens [23]. For the 40 normal controls, expressed milk was centrifuged to obtain epithelial cells which were then washed in phosphate buffered saline (PBS). For HPV and EBV, the pellet was subjected to proteinase K digestion followed by phenol extraction, washing with chloroform and alcohol precipitation. For MMTV, RNA was extracted with a Mag MAX Viral RNA isolation kit from Ambion.

Archival DNA was extracted by dewaxing paraffin embedded tissues with xylene followed by alcohol washes. The tissue was digested with proteinase K, followed by phenol extraction and alcohol precipitation. DNA integrity of all samples was confirmed by standard PCR using *β*-globin primers G073 (5′-GAAGAGCCAAGGACAGGTAC-3′) and G074 (5′-CAACTTCATCCACGTTCACC-3′).

### PCR

The sensitivity of PCR may lead to false positives due to contamination. For this reason we used both standard PCR and *in situ* PCR techniques. *In situ* PCR is less susceptible to contamination and has the important advantage of localizing the specific genetic material at the cellular level. However, *in situ* PCR remains subject to both false positive and false negative outcomes. Therefore we used stringent negative controls which included omitting DNA primers and Taq polymerase.

#### PCR screening for EBV sequences

The presence of EBV in DNA extracted from fresh frozen breast cancer specimens, normal specimens from epithelial cells in milk and archival specimens, was determined using nested PCR with primers specific for the EBNA-1 gene. Two primer sets described by Cinque *et al* [24] were used. The first round amplifying a 297 bp fragment (EB3 5′-AAGGAGGGTGGTTTGGAAAG, EB4 5′-AGACAATGGACTCCCTTAGC) and the second using a primer set that binds within the first round product generating a 209 bp fragment (EB1 5′-ATCGTGGTCAAGGAGGTTCC, EB2 5′-ACTCAATGGTGTAAGACGAC).The cycling conditions were 95°C, 3 min; followed by 35 cycles of 95°C, 30 s; 55°C, 30 s; 72°C, 30 s; and a final extension at 72°C, 5 min. Genomic DNA isolated from Raji (human Burkitt's lymphoma, EBV positive) was used as the positive control; negative controls were PCR with the omission of the DNA template as well as a reagent blank. Nested PCR was independently repeated for each sample. The amplified products were visualized on 1.5% agarose gels. PCR products, from twelve randomly selected positive PCR products from the breast tissue samples, were purified and sequenced.

#### Screening for HPV sequences

The same samples were screened for the presence of HPV by PCR using primers for a 140 base pair fragment in the E6 region of HPV 16, 18 and 33 using methods as previously described [25].

#### Screening for MMTV env sequences

The same samples were screened using the methods as previously described [19]. Standard (liquid phase) PCR was conducted at least 5 times on DNA extracted from each of 50 fresh frozen breast cancer specimens. If the outcome was always negative this analysis was recorded as negative. If the outcome was positive one or more times this analysis was recorded as positive.

#### In-situ PCR

Archival tissues on slides were washed in xylene to remove the wax followed by washes in decreasing concentrations of alcohol. The tissues were subjected to pepsin digestion with varying times of digestion that were required for different tissues. These differences were probably due to fixation procedures, which could vary in duration. The digestion was stopped in 0.1 M Tris buffer pH 8. 75 µl of PCR mix, containing inner nested PCR primers. Digoxogenin (DIG) - dUTP (0.3 nM) (Roche), was added to the tissue in a frame which was sealed. PCR cycling was the same as for standard PCR. Detection using Anti-DIG AP- Fab fragments (1 µl) (Roche) in buffer pH 7.5 followed by NBT/BCIP (2 µl) (Roche) in buffer pH 9.5 was stopped when a blue colour was observed in the cells of the cancer specimen and not in the negative control. The tissues were counterstained with eosin. Any specimens that were positive in the negative controls showed that the DNA was self priming and were unsuitable for *in-situ* work (this is probably due to fragmented DNA acting as primers). These samples were eliminated from the study. Any specimens that were negative for the three viruses were subjected to Beta-globin *in-situ* PCR to confirm the result.

### Immunohistochemistry

We used EBV latent membrane protein (LMP 1, Dako mouse clone 1–4), and EBNA-1 (monoclonal mouse anti-human EBNA-1 -1108-1, Abcam, Cambridge UK) antibodies on 28 unselected archival formalin fixed breast cancer specimens. These were a different series of specimens than the 50 fresh frozen specimens referred to above. Standard methods previously described were used [25]. Ten selected specimens of breast cancer were analysed to confirm the presence of these Epstein Barr proteins in specimens that were positive for EBV by *in situ* PCR.

### Statistics

Non-parametric statistical analyses using the R statistical system were used to analyse the difference between the age of diagnosis of breast cancer and tumour grade for virus positive compared to virus negative breast cancers.

## Results

### Screening of DNA extracted from 50 fresh frozen breast cancer specimens

**Figure 1:**
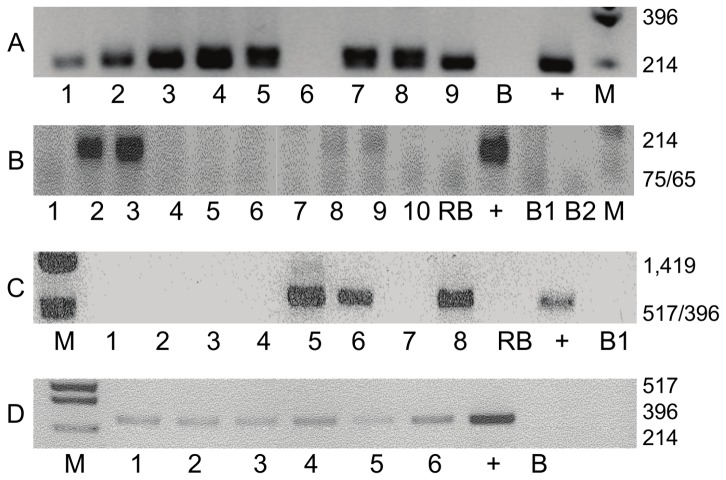
Typical nested PCR amplification for EBV, HPV and MMTV. A. EBV PCR for the second round of amplification of a 209 bp fragment from DNA extracted from 9 breast cancer specimens, B is the no DNA control, Raji DNA as a positive control (+) and M is a size ladder from Puc Hinf1. (12). B. HPV PCR for the second round amplification of a 148 bp fragment from 10 breast DNA extractions. RB is the reagent control,(+ ) is the positive control from Hela DNA containing HPV 18, B1 is a first round no DNA control for the PCR, B2 is a second round no DNA control. C. MMTV PRC for the second round of amplification of a 643 bp fragment from DNA extracted from 8 patient samples. RB is the reagent blank. (+) is the positive control of DNA extracted from mouse tails. B1 is the first round no DNA control, subjected to a second round of PCR. D. Typical *b-globin* PCR (single amplification) for 6 breast archival specimens showing the integrity of the DNA. + is a positive control from Hela DNA, B is the no DNA control.

Standard PCR was conducted at least 5 times on each sample of extracted DNA. Viral sequences were consistently detected in some samples while in others, the detection was variable (Table 1). Typical outcomes for nested PCR amplifications for EBV, HPV and MMTV are shown in Figure 1. In summary, EBV was detected in 34 (68%), HPV (all HPV 18 by sequencing) in 25 (50%) and MMTV in 39 (78%) of the 50 fresh frozen, invasive breast cancer specimens tested by standard PCR. EBV was detected in 14 (35%), HPV (all HPV 18 by sequencing) in 8 (20%) and MMTV in 13 (32%) of epithelial cells from 40 samples of milk from normal lactating women. The prevalence of EBV, HPV and MMTV sequences is significantly higher in breast cancer as compared to normal specimens (Table 1). The proportion of specimens in which no viruses were identified is significantly lower in breast cancer specimens than normal breast specimens (Table 1).

**Table 1:** Identification of EBV, HPV and MMTV viruses, either as an individual or a multiple virus, by standard PCR in 50 fresh frozen breast cancer specimens (45 invasive ductal carcinomas and 5 invasive lobular carcinomas) and epithelial cells from 40 fresh normal breast milk specimens.

	EBV	HPV	MMTV	No Virus
Breast Cancer (n = 50)	34 (68%)	25 (50%)	39 (78%)	4 (8%)
Normal breast (n = 40)	14 (35%)	8 (20%)	13 (32%)	14 (35%)
p for difference between cancer and normal	0.002	0.000	0.001	0.002

The prevalence of EBV, HPV and MMTV sequences is significantly higher in breast cancer as compared to normal specimens. The proportion of specimens in which no viruses were identified is significantly higher in normal breast specimens than in breast cancer specimens.

Multiple viruses in the same breast cancer specimen were identified frequently. The prevalence of both EBV and HPV in the same specimen, was significantly three to four fold higher in breast cancer specimens than normal control breast specimens (38% of 50 breast cancer specimens as compared to 10% of 40 normal control breast specimens).

#### Screening of DNA in 40 normal controls

Standard PCR showed variable detection of viral sequences. These variations were probably due to low viral loads. EBV was identified in 14 of 40 (35%), HPV in 8 of 40 (20%) and MMTV in 13 (32%) in epithelial cells from human milk samples (Table 1).

### In situ PCR analyses of formalin fixed breast cancer specimens

The outcomes of the *in situ* PCR analyses are shown in Table 2. The identification of these viruses in fixed specimens by *in situ* PCR is much lower than in fresh breast cancer specimens using standard liquid PCR. Multiple viral sequences were identified in 7 (26%) of the breast cancer and 4 (22%) of the normal breast specimens. The numbers of *in situ* PCR based outcomes on fixed specimens are very small and are not statistically valid.

**Table 2:** Identification of EBV, HPV and MMTV in cancer (invasive ductal carcinomas (n = 13) and ductal carcinoma *in situ* ( n = 14)) and normal specimens by *in situ* PCR on formalin fixed specimens.

	EBV	HPV	MMTV	No Virus
Ductal carcinoma in situ (n = 13)	4 (31%)	6 (46%)	3 (23%)	6 (46%)
Invasive ductal carcinoma (n = 14)	1(7% )	4 (28% )	2 (14% )	8 (57% )
Normal breast (n = 18)	6 (33%)	3 (17%)	3 (17%)	11 (61%)

**Figure 2:**
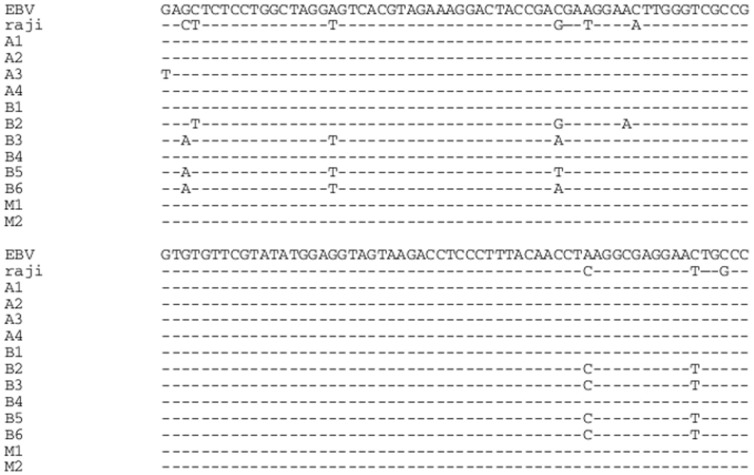
Variable sequence region of Epstein-Barr viral PCR products compared to the EBV genome (B95-8 strain). EBV, EBV genome; Raji DNA used as a positive control; A1-4 sequences are based on DNA extracts from archival formalin fixed invasive ductal carcinoma (*idc*) breast cancer specimens; B1-6 sequences are based on DNA extracts from fresh frozen *idc* breast cancer specimens. M1-2 sequences are from normal breast epithelial cell (milk) DNA extracts. The alignment of sequences demonstrates the high level of nucleotide homology between the EBV genome, the Raji EBV positive control and EBV identified by standard PCR in fixed, fresh *idc* breast cancer specimens, and normal breast specimens.

EBV, HPV and MMTV identified in the same ductal carcinoma *in situ* specimen by *in situ* PCR are shown in Figure 2. The viral sequences are located within the cancer cell nuclei. The presence of HPV associated koilocytes in these specimens is striking.

#### Sequencing results

The identity of the separate viral sequences was confirmed by automated sequencing of the PCR product from DNA extracted from selected specimens.

**Figure 3:**
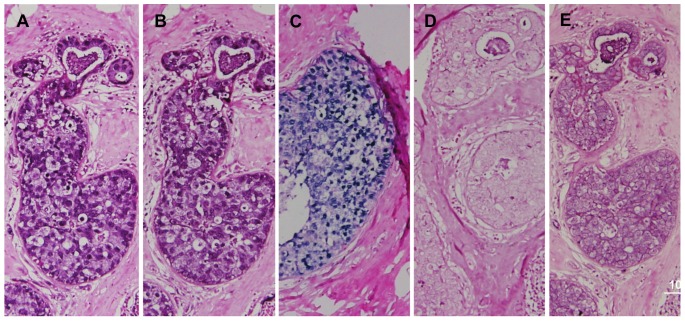
EBV, HPV and MMTV identified in the same ductal carcinoma *in situ* specimen by *in situ* PCR. A.EBV positive, B. HPV positive, C. MMTV positive, D. Negative control – no primer, E. Negative control -no Taq. HPV associated koilocytes are present in B.All photographs were taken with a 20X objective and have been cropped. Variations in colour detection can be seen when the *in-situ* PCR was done at a different time.

Sequencing of selected PCR DNA products from the various samples confirmed the presence of EBV sequences as shown in Figure 3. The alignment of sequences demonstrates the high level of nucleotide homology between the EBV genome, the Raji EBV positive control and EBV identified by standard PCR in fixed, fresh *idc* breast cancer specimens, and normal breast specimens. There is 98% to 99% homology to the EBV genome (B95-8 strain) with 6 different variants present. Variations in sequences indicate these standard PCR analyses are unlikely to be contaminated.

Some specimens were eliminated from the study because preliminary screening by *in situ* PCR with primers omitted (negative control) showed false positive outcomes. Additionally some samples were omitted if the beta-globin *in-situ* PCR gave a negative result.

Sequences confirming the presence of HPV and MMTV based on PCR products from these same breast specimens have previously been published [19], [25]. Only HPV type 18 was identified in the breast cancer specimens, however both HPV type 16 and 18 were identified in the normal breast epithelial cells from human milk samples.

**Figure 4:**
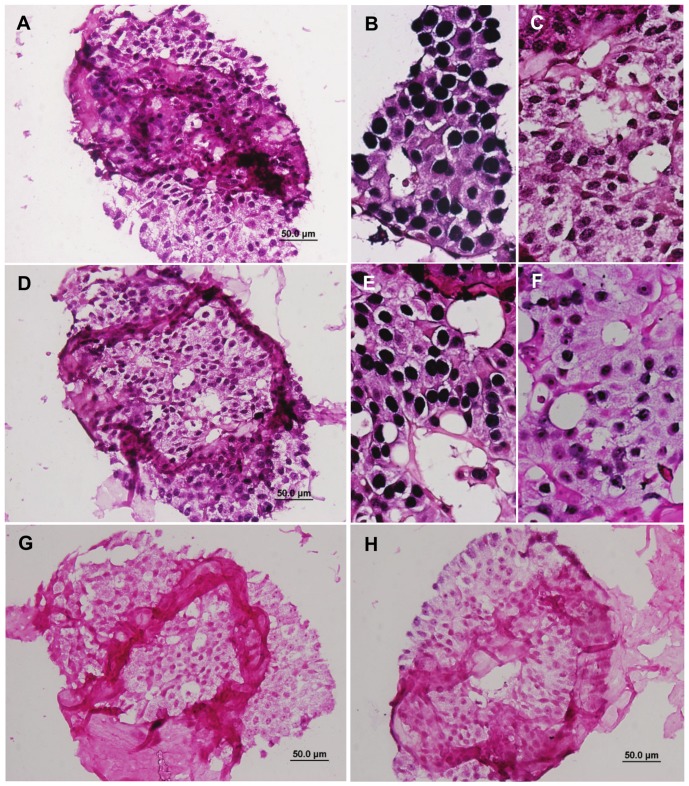
HPV and EBV identified by in situ PCR in the same breast cancer cell nuclei – Ductal carcinoma in situ. A.HPV (inner nested primers X 200). B. HPV (outer nested primers X 400). C. HPV (inner nested primers X 400). D. EBV (inner nested primers X 200). E. EBV (outer nested primers X 400). F. EBV (inner nested primers X 400). G. MMTV negative (inner nested primers X 200). H. Negative control (no primers X 200).

In Figure 4 the presence of EBV plus HPV gene sequences in the same cancer cell nuclei in the same specimen are shown. The negative result for MMTV in this same specimen indicates that random priming has not occurred. Both EBV and HPV sequences were identified in the nuclei of almost all cancer cells in the specimens shown in Figures 2 and 4. To confirm these results, the *in-situ* PCR was repeated using both the inner and outer primers for HPV and EBV as shown in Figure 4. In addition the PCR products from standard PCR conducted on these specimens were sequenced and confirmed the identity of EBV, HPV and MMTV.

### EBNA-1, LMP1, CD 15 expression

**Figure 5:**
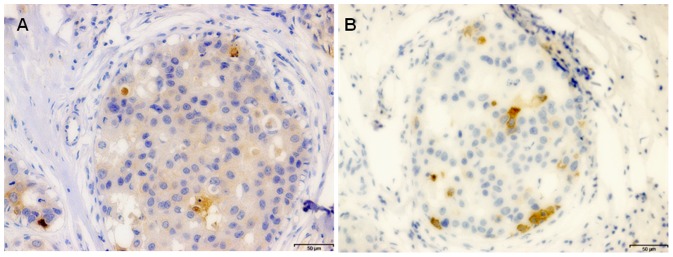
Putative Reed Sternberg cells in ductal carcinoma *in situ* (by immunohistochemistry). **Panel A.** Positive EBNA 1 expression in a putative Reed Sternberg cells. **Panel B.** Positive CD 15 expression in putative Reed Sternberg cells. It is not possible to determine whether these cells are Reed Sternberg or granuloma cells.

EBNA -1 was detected in cancer cells of 3 of 10 selected specimens. One of these three specimens was also positive for EBV identified by both standard and *in situ* PCR. EBNA 1 was expressed in only 10 to 15% of breast cancer cells in each specimen. This is shown in Figure 5. This is in contrast to the identification in virtually all of the cancer cells in the same specimens of EBV by *in situ* PCR. In addition CD15 was expressed in some cells of all 10 selected specimens. As shown in Figure 5, the EBNA positive cancer cells have histological characteristics which are similar to Reed Sternberg (RS) cells. RS cells are large multinucleated cells, which originate from B-lymphocytes. The CD 15 positive cells have similar characteristics. The EBV protein LMP1 was positively expressed in just 2 (7%) of the 28 fixed breast cancer specimens.

**Figure 6:**
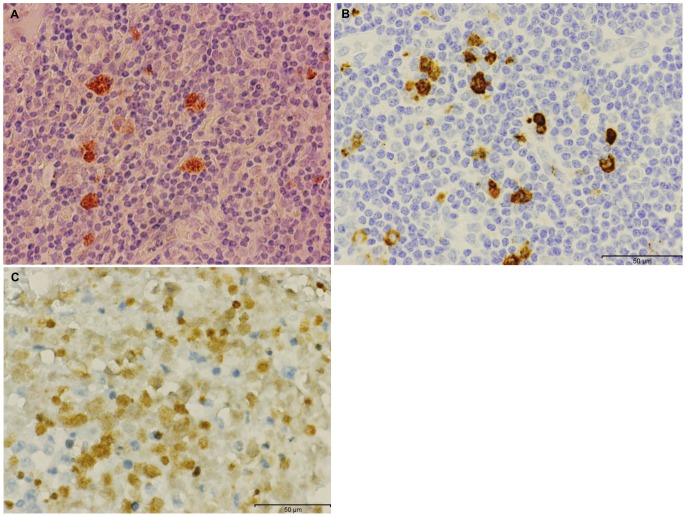
Positive EBNA, CD 15 and LMP1 expression in the same invasive ductal carcinoma breast cancer specimen. **Panel A.** Positive EBNA1 expression. **Panel B.** Positive CD15 expression. **Panel C.** Positive LMP1 expression. It is not possible to determine whether these cells are Reed Sternberg or granuloma cells.

EBNA, CD 15 and LMP1 expression were all expressed in the same invasive ductal carcinoma breast cancer specimen. These can be seen in Figure 6. It was not possible to definitively determine the identity of either the putative RS or CD15 positive cells.

### Age of patients

The average age of patients with fresh frozen breast cancer was EBV positive (57.3 years), HPV positive (54.8 years) and MMTV positive (58.2 years) and was significantly younger than patients with EBV negative (65.9 years), HPV negative (64.5 years), and MMTV negative (65.5 years) breast cancers (Table 3).

**Table 3:** EBV, HPV, and MMTV positive and negative according to patient and breast cancer characteristics.

	EBV pos	EBV neg	p value	HPV pos	HPV neg	p value	MMTV pos	MMTV neg	p value
Age-years	57.3	65.9	0.001	54.8	64.5	0.001	58.2	65.5	0.001
**Grade**									
I	2	3	0.177	4	1	0.309	3	2	0.187
II	10	7		7	10		11	6	
III	18	5		12	11		20	3	
**ER**									
Neg	7	2		4	6		8	2	
Pos	26	14		19	20		29	9	
**PR**									
Neg	13	2		7	8		14	1	
Pos	22	12		16	18		24	10	
**HER**									
Neg	24	11		17	18		27	8	
Pos	3	0		2	1		2	1	
**P53**									
Neg	6	0		4	2		5	1	
Pos	26	17		20	24		34	10	

These data are based on DNA extracts from 50 fresh frozen invasive breast tumours. There are small variations in the numbers of specimens analysed for each virus because of inadequate outcomes of some analyses based on standard PCR. The p values based on the Chi – square test are for differences in the presence of viruses between grades of breast cancer. The p values are not included for data based on immunohistochemistry because of low numbers.

These data indicate: (i) patients with HPV, EBV and MMTV positive breast cancer are significantly younger than patients with viral negative breast cancer, (ii) there is a non-significant increase in grade of EBV and MMTV positive breast cancer, (iii) the expression of apoptopic p53 protein is not inhibited in HPV positive breast cancer as it is in HPV positive cervical cancer.

### Breast cancer grade

There is a non-significant increase in grade of EBV and MMTV positive invasive breast cancer specimens (Table 3).

### Estrogen and progesterone receptor, HER and p53expression

There are no obvious differences in estrogen and progesterone receptor expression nor HER2 expression between viral positive and negative fresh breast cancer specimens (Table 3).

## Discussion

We have demonstrated that EBV, HPV, and MMTV viral sequences may all be present as individual or multiple viruses in human breast cancer and in some normal breast tissues and normal breast milk epithelial cells. These three viruses appear to be present in a significantly higher proportion of breast cancers as compared to breast epithelial cells in human milk from normal lactating women.

The identification of EBV in this current study, using standard PCR, *in situ* PCR, and immunohistochemistry, is consistent with the findings of others (as listed in Table S1). However, it is possible that some previous findings may have been exaggerated by the phenomenon of lytic viral replication as identified by Huang *et al* [26]. False positive outcomes due to lytic viral replication are confined to standard PCR. In this current study we have shown by both *in situ* PCR and EBNA and LMP 1 expression that EBV may be present in individual cancer cells. This observation is consistent with findings by others, [9], [10], [27], [28]. Therefore our findings are likely to be accurate.

The viral load of HPV, MMTV and EBV in breast cancer appears to be extremely low. In a study of HPV in breast cancer that had developed in Japanese women, Kahn et al [29] estimated the viral load of HPV in cervical cancer was 4,000 fold greater than in breast cancer. It is also known that retroviruses such as MMTV may have only one integrated viral genome per host cell [30]. Accordingly, these viruses may only be detected by methods which involve amplification such as standard and *in situ* PCR. It was the development of *in situ* PCR that revolutionized the early treatment of patients infected with HIV, as this technique was sufficiently sensitive to identify virally infected cells well before the development of symptoms. These important technical issues are discussed in detail by Nuovo [30] and Joshi and Buehring [31]. Joshi and Buehring in their review of these three viruses and breast cancer have shown that investigations based on in situ hybridisation offer mostly negative outcomes whereas PCR techniques, often in the same specimens, consistently offer positive outcomes [31].

While EBV may not be oncogenic when present in isolation in normal breast tissues, it may have oncogenic influences if and when it collaborates with other viruses. Recently, Hagensee et al [32] have experimentally demonstrated an interaction between HPV and EBV (whole viruses) in vivo, and an interaction between HPV and EBV oncoprotein, LMP1 (in absence of EBV genome and other EBV proteins) in vitro (leading to reduced apoptosis). This intriguing evidence supports the notion that these viruses may collaborate in oncogenic processes.

Only HPV types 18 have been identified in Australian breast cancers. This has been independently shown by both our group and the Antonsson group in Brisbane [25], [33]. This is in contrast to the identification of a range of HPV types in breast cancer in other countries [1]. As HPV type 18 is tropic to glandular, as distinct from squamous epithelial cells, we speculate that despite HPV type 16 being present in normal Australian breast epithelial cells, HPV type 18 dominates in Australian breast cancer epithelial cells.

The striking presence of HPV associated koilocytes in the dcis specimens has previously been reported [34]. Koilocytes are an indication of early oncogenic influences of HPV and in particular the influence of active HPV E5 and E6 proteins [35].

### Age of virus positive patients

The observation that patients with HPV positive breast cancers at age of diagnosis are younger than women with HPV negative breast cancers is consistent with other studies [18]. This observation is also consistent with the hypothesis that women who have HPV associated cervical pathology and who later develop HPV positive breast cancer at a young age may have sexually transmitted HPV [18]. As HPVs have been identified in breast nipples and also in white blood cells, it is possible that HPVs may have been transmitted to the breast by physical contact or through the bloodstream [36].

### Breast cancer grade

The non significant trend that the presence of EBV is associated with higher grade breast cancer is consistent with the recent observations by Mazouni et al [37]. In a prior investigation of a different series of breast tumors we observed an increased prevalence of MMTV associated with progression from normal to increased grades of breast cancer [38]. However, recent studies by the Bevilacqua group in Italy suggest the opposite, namely that the prevalence of MMTV decreases as the grade of breast cancer increases [21]. This latter finding may be due to the use of more reliable *in situ* PCR techniques by Bevilacqua *et al*.

### Estrogen and progesterone receptor, HER and p53 expression

There were no obvious associations between the presence of single or multiple viruses in breast cancer and expression of ER, PR, HER and p53. The number of cases is too small to make confident implications.

### EBNA, LMP, CD 15 expression and Reed Sternberg cells

The expression of EBNA1 in only 10 to 15% of cancer cells in breast cancer specimens is consistent with the previous findings of Bonnet et al [8] and Joshi et al [28]. Some of the EBNA positive cancer cells have histological characteristics which are similar to Reed Sternberg (RS) cells. The CD 15 positive cells in these same specimens, have similar characteristics. While it was not possible to definitively determine the identity of these cells, it may be that EBV positive lymphocytes have infiltrated breast tissues and transmitted EBV to breast epithelial cells. Some of these EBV positive lymphocytes have characteristics of RS cells.

The very limited detection of LMP1 protein in these series of breast cancers is consistent with the lack of expression reported in non-Hodgkin's lymphomas or carcinomas [39].

The influence of these different three viruses may be reflected in the differences in breast cancer morphology and phenotypes. We have previously shown that MMTV positive breast cancers may be histologically similar to MMTV associated mouse mammary tumours [19].

EBV, HPV and MMTV have been identified in a range of body organs but with greatly varying viral loads [1]. Accordingly these viruses may be cell type specific and not organ specific. HPV has been identified in genital cancers, cancers of the head and neck, breast and prostate cancers and even bowel and lung cancers [40]. EBV has been associated with Hodgkins lymphoma, Burkitts lymphoma and nasopharyngeal cancer. MMTV has been identified in human breast, prostate and liver tissues [20], [41].

We conclude that (i) EBV, HPV and MMTV gene sequences are present and co-exist in many human breast cancers, (ii) the presence of these viruses in breast cancer is associated with young age of diagnosis and possibly an increased grade of breast cancer and (iii) EBV and HPV may collaborate in some breast cancers. These three viruses may each have oncogenic roles in human breast cancer.

## Supporting Information

Table S1 **Published studies of Epstein Barr virus in breast cancer.**
(DOCX)
