# Supplementary material for: Epstein-Barr Virus, Human Papillomavirus and Mouse Mammary Tumour Virus as Multiple Viruses in Breast Cancer
Source: PLoS One. 2012 Nov 19;7(11):e48788. doi: 10.1371/journal.pone.0048788 (PMC3501510; doi:10.1371/journal.pone.0048788)
Supplement: Table S1 — Published studies of Epstein Barr virus in breast cancer. (DOCX) [file pone.0048788.s001.docx]

**Table S1. Published studies of Epstein Barr virus in breast cancer.**

| First author, year (Reference number) | Population | Specimens | Assay | % EBV positive cancer specimens ,(number) | % EBV positive normal control specimens, (number) |
| --- | --- | --- | --- | --- | --- |
| Gaffey 1993 (1) | US | Fixed | PCR^1^ | 0% (35) | - |
| Horiuchi 1994 (2) | Japan | Fixed | PCR | 66% (3) | - |
| Luqmani 1995 (3) | UK | Fixed | PCR | 54% (28) | - |
| Lespagnard 1995(4) | Belgium | Fixed | PCR | 0% (10) | - |
| Labrecque 1995 (5) | UK | Fixed | PCR | 21% (91) | 0% (21) |
| Chu 1998 (6) | Taiwan | Fixed | IHC^2^ | 0% (60) | - |
| Glaser 1998 (7) | US | Fixed | ISH^3^ | 0% (107) | - |
| Bonnet 1999 (8) | France | Frozen | PCR,IHC | 51% (100) | Adjacent   10% (30) |
| Brink 2000 (9) | Holland | Frozen | PCR | 21% (24) | - |
| McCall 2001 (10) | US | Fixed | PCR | 2% (115) | - |
| Dadmanesh 2001 (11) | Italy | Fixed | ISH | 0% (4) | - |
| Kijima 2001 (12) | Japan | Fixed | ISH | 0% (61) | - |
| Fina 2001 (13) | N. Africa, Europe | Fixed, frozen | PCR, ISH | 32% (509) | - |
| Chu 2001 (14) | US | Fixed, frozen | PCR | 10% (48) | - |
| Grinstein 2002 (15) | US | Fixed | PCR | 100% (33) | - |
| Deshpande 2002 (16) | US | Fixed | ISH, IHC | 0% (43) | - |
| Herrman 2003 (17) | Germany | Fixed | PCR | 7% (59) | - |
| Xue 2003 (18) | UK | Fixed, frozen | PCR | 40% (15) | - |
| Murray 2003 (19) | UK | Fixed | PCR | 21% (153) | - |
| Preciado 2005 (20) | US & Argentina | Fixed | PCR | 35% (69) | Adjacent 0% (8) |
| Perrigoue 2005 (21) | US | Fixed | PCR | 0% (45) | - |
| Kalkan 2005 (22) | Turkey | Fixed | PCR | 23% (57) | 35% (55) |
| Tsai 2005 (23) | Taiwan | Frozen | PCR | 45% (62) | - |
| Thorne 2005 (24) | US | Fixed | PCR | 7% (55) | - |
| Arbach 2006 (25) | France | Frozen | PCR | 46% (95) | - |
| Perkins 2006 (26) | US | Fresh | PCR | 46% (24) | - |
| Fawzy 2008 (27) | Egypt | Fixed | PCR | 20% (40) | 0 % (20) |
| Trabelsi 2008 (28) | Tunisia | Fixed | IHC, ISH | 0% (36) | - |
| Joshi 2009 (29) | India | Fixed | IHC | 55% (51) | - |
| Mazouni 2010 (30) | France | Frozen | PCR | 33% (196) | - |
| Lorenzetti 2010 (31) | Argentina | Fixed | PCR/IHC/ ISH | 31% (71) | - |
| Kadivar 2011 (32) | Iran | Fixed | PCR/IHC | 0% (100) | 0% (42) |

^1^PCR = polymerase chain reaction, ^2^IHC = immunohistochemistry, ^3^ISH = *in situ* hybridisation.

**Supplementary Table 1 References**

1. Gaffey MJ, Frierson HF Jr, Mills SE, Boyd JC, Zarbo RJ et al. (1993) [Medullary carcinoma of the breast. Identification of lymphocyte subpopulations and their significance.](http://www.ncbi.nlm.nih.gov/pubmed/8302815?ordinalpos=2&itool=EntrezSystem2.PEntrez.Pubmed.Pubmed_ResultsPanel.Pubmed_DefaultReportPanel.Pubmed_RVDocSum) Mod Pathol 6:721-728.

2. K, Mishima K, Ohsawa M, Aozasa K. (1994) [Carcinoma of stomach and breast with lymphoid stroma: localisation of Epstein-Barr virus.](http://www.ncbi.nlm.nih.gov/pubmed/8063937?ordinalpos=73&itool=EntrezSystem2.PEntrez.Pubmed.Pubmed_ResultsPanel.Pubmed_DefaultReportPanel.Pubmed_RVDocSum) J Clin Pathol 47:538-540.

3. Luqmani Y A, Shousha S. (1995)Presence of Epstein-Barr virus in breast carcinoma. Int J Oncol 6:899-903.

4. Lespagnard L, Cochaux P, Larsimont D, Degeyter M, Velu T et al. (1995) Absence of Epstein-Barr virus in medullary carcinoma of the breast as demonstrated by immunophenotyping, in situ hybridization and polymerase chain reaction. Am J Clin Pathol 103:449-452.

5. [Labrecque LG](http://www.ncbi.nlm.nih.gov/sites/entrez?Db=pubmed&Cmd=Search&Term=%22Labrecque%20LG%22%5BAuthor%5D&itool=EntrezSystem2.PEntrez.Pubmed.Pubmed_ResultsPanel.Pubmed_DiscoveryPanel.Pubmed_RVAbstractPlus), [Barnes DM](http://www.ncbi.nlm.nih.gov/sites/entrez?Db=pubmed&Cmd=Search&Term=%22Barnes%20DM%22%5BAuthor%5D&itool=EntrezSystem2.PEntrez.Pubmed.Pubmed_ResultsPanel.Pubmed_DiscoveryPanel.Pubmed_RVAbstractPlus), [Fentiman IS](http://www.ncbi.nlm.nih.gov/sites/entrez?Db=pubmed&Cmd=Search&Term=%22Fentiman%20IS%22%5BAuthor%5D&itool=EntrezSystem2.PEntrez.Pubmed.Pubmed_ResultsPanel.Pubmed_DiscoveryPanel.Pubmed_RVAbstractPlus), [Griffin BE](http://www.ncbi.nlm.nih.gov/sites/entrez?Db=pubmed&Cmd=Search&Term=%22Griffin%20BE%22%5BAuthor%5D&itool=EntrezSystem2.PEntrez.Pubmed.Pubmed_ResultsPanel.Pubmed_DiscoveryPanel.Pubmed_RVAbstractPlus). (1995) Epstein-Barr virus in epithelial cell tumours: a breast cancer study. Cancer Res 55:39-45.

6. Chu JS, Chen CC, Chang KJ. (1998)[In situ detection of Epstein-Barr virus in breast cancer.](http://www.ncbi.nlm.nih.gov/pubmed/9500191?ordinalpos=4&itool=EntrezSystem2.PEntrez.Pubmed.Pubmed_ResultsPanel.Pubmed_DefaultReportPanel.Pubmed_RVDocSum) Cancer Lett 124:53-57.

7: Glaser SL, Ambinder RF, DiGiuseppe JA, Horn-Ross PL, Hsu JL. (1998) [Absence of Epstein-Barr virus EBER-1 transcripts in an epidemiologically diverse group of breast cancers.](http://www.ncbi.nlm.nih.gov/pubmed/9466655?ordinalpos=10&itool=EntrezSystem2.PEntrez.Pubmed.Pubmed_ResultsPanel.Pubmed_DefaultReportPanel.Pubmed_RVDocSum) Int J Cancer 75:555-558.

8. Bonnet M, Guinebretiere J-M, Kremmer E, Grunewald V, Benhamou E et al. (1999) Detection of Epstein-Barr virus in invasive breast cancer. J Natl Cancer Inst91:1376-1381.

9. Brink AA, van den Brule AJC, van Diest, Meijer CJLM. (2000) Detection of Epstein Barr virus in breast cancer. J Natl Cancer Inst 92:655-656.

10. McCall SA, Lichy JH, Bijwaard KE, Aguilera NS, Chu WS et al. (2000) [Epstein-Barr virus detection in ductal carcinoma of the breast.](http://www.ncbi.nlm.nih.gov/pubmed/11208885?ordinalpos=2&itool=EntrezSystem2.PEntrez.Pubmed.Pubmed_ResultsPanel.Pubmed_DefaultReportPanel.Pubmed_RVDocSum) J Natl Cancer Inst 93:148-150.

11. Dadmanesh F, Peterse JL, Sapino A, Fonelli A, Eusebi V:. (2001) [Lymphoepithelioma-like carcinoma of the breast: lack of evidence of Epstein-Barr virus infection.](http://www.ncbi.nlm.nih.gov/pubmed/11135047?ordinalpos=3&itool=EntrezSystem2.PEntrez.Pubmed.Pubmed_ResultsPanel.Pubmed_DefaultReportPanel.Pubmed_RVDocSum) Histopathol 38:54-61.

12. Kijima Y, Hokita S, Takao S, Baba M, Natsugoe S et al. (2001) [Epstein-Barr virus involvement is mainly restricted to lymphoepithelial type of gastric carcinoma among various epithelial neoplasms.](http://www.ncbi.nlm.nih.gov/pubmed/11468737?ordinalpos=25&itool=EntrezSystem2.PEntrez.Pubmed.Pubmed_ResultsPanel.Pubmed_DefaultReportPanel.Pubmed_RVDocSum) J Med Virol 64:513-518.

13. Fina F, Romain S, Ouafik L, Palmari J, Ben Ayed F et al. (2001) [Frequency and genome load of Epstein-Barr virus in 509 breast cancers from different geographical areas.](http://www.ncbi.nlm.nih.gov/pubmed/11259092?ordinalpos=3&itool=EntrezSystem2.PEntrez.Pubmed.Pubmed_ResultsPanel.Pubmed_DefaultReportPanel.Pubmed_RVDocSum) Br J Cancer 84:783-790.

14. Chu PG, Chang KL, Chen YY, Chen WG, Weiss LM. (2001) [No significant association of Epstein-Barr virus infection with invasive breast carcinoma.](http://www.ncbi.nlm.nih.gov/pubmed/11485915?ordinalpos=3&itool=EntrezSystem2.PEntrez.Pubmed.Pubmed_ResultsPanel.Pubmed_DefaultReportPanel.Pubmed_RVDocSum) Am J Pathol 159:571-578.

15. Grinstein S, Preciado MV, Gattuso P, Chabay PA, Warren WH et al. (2002) Demonstration of Epstein-Barr virus in carcinomas of various sites. Cancer Res 62:4876-4878.

16. Deshpande CG, Badve S, Kidwai N, Longnecker R. (2002) [Lack of expression of the Epstein-Barr Virus (EBV) gene products, EBERs, EBNA1, LMP1, and LMP2A, in breast cancer cells.](http://www.ncbi.nlm.nih.gov/pubmed/12218080?ordinalpos=2&itool=EntrezSystem2.PEntrez.Pubmed.Pubmed_ResultsPanel.Pubmed_DefaultReportPanel.Pubmed_RVDocSum) Lab Invest 82:1193-1199.

17. Herrmann K, Niedobitek G. (2003) Lack of evidence for an association of Epstein-Barr virus infection with breast carcinoma. Breast Cancer Res 5:R13-7.

18. Xue SA, Lampert IA, Haldane JS, Bridger JE, Griffin BE. (2003) Epstein-Barr virus gene expression in human breast cancer: protagonist or passenger. Br J Cancer 89:113-119.

19. Murray PG, Lissauer D, Junying J, Davies G, Moore S et al. (2003) [Reactivity with a monoclonal antibody to Epstein-Barr virus (EBV) nuclear antigen 1 defines a subset of aggressive breast cancers in the absence of the EBV genome.](http://www.ncbi.nlm.nih.gov/pubmed/12727860?ordinalpos=10&itool=EntrezSystem2.PEntrez.Pubmed.Pubmed_ResultsPanel.Pubmed_DefaultReportPanel.Pubmed_RVDocSum) Cancer Res 63:2338-2343.

20. Preciado MV, Chabay PA, De Matteo EN, Gonzalez P, Grinstein S et al. (2005) [Epstein-Barr virus in breast carcinoma in Argentina.](http://www.ncbi.nlm.nih.gov/pubmed/15737034?ordinalpos=2&itool=EntrezSystem2.PEntrez.Pubmed.Pubmed_ResultsPanel.Pubmed_DefaultReportPanel.Pubmed_RVDocSum) Arch Pathol Lab Med 129:377-381.

21. [Perrigoue JG](http://www.ncbi.nlm.nih.gov/sites/entrez?Db=pubmed&Cmd=Search&Term=%22Perrigoue%20JG%22%5BAuthor%5D&itool=EntrezSystem2.PEntrez.Pubmed.Pubmed_ResultsPanel.Pubmed_DiscoveryPanel.Pubmed_RVAbstractPlus), [den Boon JA](http://www.ncbi.nlm.nih.gov/sites/entrez?Db=pubmed&Cmd=Search&Term=%22den%20Boon%20JA%22%5BAuthor%5D&itool=EntrezSystem2.PEntrez.Pubmed.Pubmed_ResultsPanel.Pubmed_DiscoveryPanel.Pubmed_RVAbstractPlus), [Friedl A](http://www.ncbi.nlm.nih.gov/sites/entrez?Db=pubmed&Cmd=Search&Term=%22Friedl%20A%22%5BAuthor%5D&itool=EntrezSystem2.PEntrez.Pubmed.Pubmed_ResultsPanel.Pubmed_DiscoveryPanel.Pubmed_RVAbstractPlus), [Newton MA](http://www.ncbi.nlm.nih.gov/sites/entrez?Db=pubmed&Cmd=Search&Term=%22Newton%20MA%22%5BAuthor%5D&itool=EntrezSystem2.PEntrez.Pubmed.Pubmed_ResultsPanel.Pubmed_DiscoveryPanel.Pubmed_RVAbstractPlus), [Ahlquist P](http://www.ncbi.nlm.nih.gov/sites/entrez?Db=pubmed&Cmd=Search&Term=%22Ahlquist%20P%22%5BAuthor%5D&itool=EntrezSystem2.PEntrez.Pubmed.Pubmed_ResultsPanel.Pubmed_DiscoveryPanel.Pubmed_RVAbstractPlus) et al. (2005) Lack of association between EBV and breast carcinoma. Cancer Epidemiol Biomarkers Prev 14:809-814.

22. Kalkan A, Ozdarendeli A, Bulut Y, Yekeler H, Cobanoglu B et al. (2005) Investigation of Epstein-Barr virus DNA in formalin-fixed and paraffin- embedded breast cancer tissues. Med Princ Pract 14:268-271.

23. Tsai JH, Tsai CH, Cheng MH, Lin SJ, Xu FL et al. (2005) Association of viral factors with non-familial breast cancer in Taiwan by comparison with non-cancerous, fibroadenoma and thyroid tumour tissues. J Med Virol 75:276-281.

24. Thorne LB, Ryan JL, Elmore SH, Glaser SL, Gulley ML. (2005) [Real-time PCR measures Epstein-Barr Virus DNA in archival breast adenocarcinomas.](http://www.ncbi.nlm.nih.gov/pubmed/15714061?ordinalpos=2&itool=EntrezSystem2.PEntrez.Pubmed.Pubmed_ResultsPanel.Pubmed_DefaultReportPanel.Pubmed_RVDocSum) Diagn Mol Pathol 14:29-33.

25. [Arbach H](http://www.ncbi.nlm.nih.gov/sites/entrez?Db=pubmed&Cmd=Search&Term=%22Arbach%20H%22%5BAuthor%5D&itool=EntrezSystem2.PEntrez.Pubmed.Pubmed_ResultsPanel.Pubmed_DiscoveryPanel.Pubmed_RVAbstractPlus), [Viglasky V](http://www.ncbi.nlm.nih.gov/sites/entrez?Db=pubmed&Cmd=Search&Term=%22Viglasky%20V%22%5BAuthor%5D&itool=EntrezSystem2.PEntrez.Pubmed.Pubmed_ResultsPanel.Pubmed_DiscoveryPanel.Pubmed_RVAbstractPlus), [Lefeu F](http://www.ncbi.nlm.nih.gov/sites/entrez?Db=pubmed&Cmd=Search&Term=%22Lefeu%20F%22%5BAuthor%5D&itool=EntrezSystem2.PEntrez.Pubmed.Pubmed_ResultsPanel.Pubmed_DiscoveryPanel.Pubmed_RVAbstractPlus), [Guinebretière JM](http://www.ncbi.nlm.nih.gov/sites/entrez?Db=pubmed&Cmd=Search&Term=%22Guinebreti%C3%A8re%20JM%22%5BAuthor%5D&itool=EntrezSystem2.PEntrez.Pubmed.Pubmed_ResultsPanel.Pubmed_DiscoveryPanel.Pubmed_RVAbstractPlus), [Ramirez V](http://www.ncbi.nlm.nih.gov/sites/entrez?Db=pubmed&Cmd=Search&Term=%22Ramirez%20V%22%5BAuthor%5D&itool=EntrezSystem2.PEntrez.Pubmed.Pubmed_ResultsPanel.Pubmed_DiscoveryPanel.Pubmed_RVAbstractPlus) et al. (2006) Epstein-Barr virus (EBV) genome and expression in breast cancer tissue: effect of EBV infection of breast cancer cells on resistance to paclitaxel (Taxol). J Virol 80:845-853.

26. Perkins RS, Sahm K, Marando C, Dickson-Witmer D, Pahnke GR et al. (2006) Analysis of Epstein-Barr virus reservoirs in paired blood and breast cancer primary biopsy specimens by real time PCR. Breast Cancer Res 8:R70.

27. Fawzy S, Sallam M, Awad NM. (2008) [Detection of Epstein-Barr virus in breast carcinoma in Egyptian women.](http://www.ncbi.nlm.nih.gov/pubmed/18258188?ordinalpos=1&itool=EntrezSystem2.PEntrez.Pubmed.Pubmed_ResultsPanel.Pubmed_DefaultReportPanel.Pubmed_RVDocSum) Clin Biochem 41:486-492.

28. Trabelsi AR, Stita W, Mokni W, Mourou A, Korbi S. (2008) Detection of Epstein Barr virus in breast cancers with lymphoid stroma. Ann Biol Clin 66:59-62.

29. [Joshi D](http://www.ncbi.nlm.nih.gov/pubmed?term=%22Joshi%20D%22%5BAuthor%5D), [Quadri M](http://www.ncbi.nlm.nih.gov/pubmed?term=%22Quadri%20M%22%5BAuthor%5D), [Gangane N](http://www.ncbi.nlm.nih.gov/pubmed?term=%22Gangane%20N%22%5BAuthor%5D), [Joshi R](http://www.ncbi.nlm.nih.gov/pubmed?term=%22Joshi%20R%22%5BAuthor%5D), [Gangane N](http://www.ncbi.nlm.nih.gov/pubmed?term=%22Gangane%20N%22%5BAuthor%5D). (2009) Association of Epstein Barr virus infection (EBV) with breast cancer in rural Indian women. PLoS One 4:e8180.

30. Mazouni C, Fina F, Romain S, Ouafik L, Bonnier P et al. (2011) [Epstein-Barr virus as a marker of biological aggressiveness in breast cancer.](http://www.ncbi.nlm.nih.gov/pubmed/21179039) Br J Cancer 104:332-337.

31. Lorenzetti MA, De Matteo E, Gass H, Martinez Vazquez P et al. (2011) [Characterization of Epstein Barr virus latency pattern in Argentine breast carcinoma.](http://www.ncbi.nlm.nih.gov/pubmed/21042577) PLoS One 5:e13603.

32. Kadivar M, Monabati A, Joulaee A, Hosseini N. (2011) [Epstein-Barr Virus and Breast Cancer: Lack of Evidence for an Association in Iranian Women.](http://www.ncbi.nlm.nih.gov/pubmed/21207256) Pathol Oncol Res 17:489-492.
